# Women and household responsibilities under COVID-19 lockdowns: many steps backwards in implementing SDG 5 in South Africa

**DOI:** 10.3389/fgwh.2025.1654642

**Published:** 2025-11-25

**Authors:** Godwell Nhamo, Malebajoa Anicia Maoela

**Affiliations:** 1 Exxaro Chair in Climate and Sustainability Transitions, University of South Africa, Pretoria, South Africa; 2 Holistic Climate Change Impact Area, Council for Scientific and Industrial Research, Pretoria, South Africa

**Keywords:** COVID-19, SDG 5, SDG 3, SDG 4, stakeholders, women, girls, unpaid work

## Abstract

Debates continue regarding the impacts of COVID-19 on women and girls. Lockdowns increased home-based responsibilities, causing work overloads and trauma. This study investigated the impact of COVID-19 lockdowns on women's household responsibilities and caregiving roles in Limpopo Province, assessing how these effects differed between urban and rural settings. Guided by the 2030 Agenda for Sustainable Development and key SDGs, including SDG 5 (Gender), SDG 4 (Quality Education), and SDG 6 (Water and Sanitation), the research utilized a quantitative survey-based approach, including household and teacher surveys. A total of 4,571 completed household surveys and 226 teacher surveys were conducted between October 2022 and March 2023. The study explored various aspects of gendered experiences during the pandemic, including water access, sanitation, education disruptions, and the increased caregiving burdens placed on women. Descriptive and inferential statistical analyses, including ANOVA tests, were conducted to examine the relationships between household dynamics, water access, and gendered responsibilities. The data revealed significant gender disparities, with women disproportionately impacted by inadequate water, unreliable sanitation facilities, and the added burdens of unpaid caregiving. The research highlighted the increased vulnerability of the girl child, particularly regarding school dropouts and unwanted pregnancies. The paper recommends gendered perspectives in pandemic responses and age-sensitive policies for resilience. It also urges decision-makers to recognize the long-term impacts of COVID-19 on achieving SDG 5 and related goals, such as SDG 3.

## 1 Introduction

The COVID-19 pandemic exposed and exacerbated existing gender inequalities particularly affecting the lives of women and girls across the globe (1, 2). In South Africa, the pandemic intensified the unequal distribution of unpaid domestic work, increased the incidence of gender-based violence, and placed a disproportionate mental and emotional burden on women (2). These impacts intersect with efforts to promote gender equality, especially those outlined in the United Nations Development Goal 5 (SDG 5) which is dedicated to achieving “gender equity and empower all women and girls” [(3): 14&18].

In Limpopo Province, the COVID-19 pandemic posed significant health and socio-economic challenges, exacerbated by the region's predominantly rural character, limited healthcare infrastructure, and high poverty rates (4). During the national lockdowns, Limpopo experienced mobility restrictions, temporary school closures, and disrupted service delivery, further straining economically vulnerable households (5). The provincial government, in line with national directives, implemented public health measures including mandatory mask-wearing, social distancing, and the distribution of relief grants to vulnerable populations (6). However, access to healthcare and mental health services remained limited in remote areas, heightening the impact of the pandemic on women and girls, who often bear the burden of caregiving, food provision, and household responsibilities (7).

Fisseha et al. (8) realises that the impact of COVID-19 went beyond the disease itself. In fact, Ronnie et al. (9) is of the view that it was a time of great stress, while Chauhan (10) contents that the environment surrounding COVID-19 is not gender neutral. Some of the impacts observed for women (8) include the increased burden of unpaid labour, scaled up gender-based violence in the households, and under-age marriages. Orellana et al. (11) adds that household food-related tasks like shopping and cooking became increased as there was a need to plan for such an activity. Such impacts led to additional mental health suffering (8), limited access to sexual and reproductive health services, as well as reduced employment opportunities and incomes. Drawing lessons from the USA, Liu and Gan (12) discover that unprecedented closures of large-scale childcare facilities resulted in dramatic increases in the burden of childcare at home. This challenge was also disproportionately addressed by women more than men (13), although this gendered effect was only reflected among parents with lower educational attainments and family incomes (12).

Tracking the housework re-allocation between gender and generations during the pandemic lockdowns in China, Wang (14) analysed 1,669 respondents from a survey, and 100 interviews. The research sought to examine changes in domestic labour patterns and the underlying reasons using homebound and job-free individuals whose employment status remained constant. The results show that men increase their uptake in grocery shopping, but reduce their cooking, cleaning, and laundry roles. The reasons given for these changes converged around the “doing gender” theory. Women in full-time employment ended up with more time to perform household chores, not to say their male counterparts did not have time on their hands. The findings challenge the notion that economic factors are the dominant drivers of the gender-based division of housework. Instead, the traditional ingrained gender norms that provided the continued framework that determined domestic roles remain. Household food-related tasks were also chosen by Orellana et al. (11) as deliberated earlier.

Expanding on mental health matters, and drawing from South Africa, Kopylova et al. (15) highlights that a significant proportion of women were already experiencing symptoms of depression prior to the onset of COVID-19, particularly those facing socio-economic hardship, unemployment, or caregiving burdens. Their findings point to an alarming pre-pandemic mental health baseline, which was further exacerbated by the pandemic's social and economic disruptions. While the study focused on national trends, these issues are particularly relevant to provinces like Limpopo, where high poverty rates, limited access to healthcare (4, 5), and rural isolation compound existing vulnerabilities. In Limpopo, women are often primary caregivers and are more likely to experience food insecurity and limited psychosocial support, increasing their susceptibility to depression and anxiety during crisis periods. Social isolation measures, including prolonged lockdowns and school closures have been linked to heightened psychological distress, especially among caregivers tasked with childcare under constrained household conditions (16). Evidence also suggests that living in a food-insecure household or caring for dependents for extended hours sometimes between 13 and 24 h a day, may increase the risk of mental health challenges, including anxiety and depression (17). Conversely, access to social protection mechanisms, such as government grants, and adherence to public health measures like mask-wearing, have been associated with reduced psychological strain during the pandemic (18).

Building on these perspectives, this study examines how COVID-19 lockdowns affected women's and girls’ household responsibilities in the Limpopo province of South Africa. Three objectives are outlined as follows: (1) To evaluate perceptions of how time was spent by women at home during COVID-19 lockdowns from their own point of view, (2) To determine male perspectives on how women spent their time at home during COVID-19 lockdowns, (3) To establish whether age and other socio-economic parameters played a role in responsibilities and time spent in the house by women during COVID-19 lockdowns. This work brings additional value to the body of literature broadly but, more specifically, to work pitched at municipal and household level in South Africa. The findings remain valuable in informing the current and future policy related to reducing pandemic and disaster risk.

## 2 Literature review

This section presents the relevant literature associated with women's household responsibilities during COVID-19 lockdown restrictions. It also focuses on highlighting potential pressure points including the role played by water, sanitation and hygiene (WASH). Perspectives on teen pregnancies and school dropouts are also discussed. While further details will be unravelled in the following sub-sections, Haney and Barber (19) remind us that “For many years, scholars have directed our attention to the gender gap in domestic labour. Even when women engage in paid employment, they nevertheless perform the majority of the household labour in most wealthy countries”.

### 2.1 Potential pressure points at home

In dealing with the responsibilities and time that women spend in their homes during the COVID-19 pandemic, it becomes essential to understand the pressure points that emerged and intensified in this period. In the context of this study, pressure points refer to critical areas of stress or vulnerability within households that became particularly pronounced during the pandemic. These points often revealed underlying social and infrastructural challenges, especially in low-resource settings. This paper focuses on three interconnected pressure points: water, sanitation and hygiene (WASH); increased domestic responsibilities; and the rise in teen pregnancies and school dropouts.

These areas were not selected arbitrarily, but rather because they intersect at the household level and reflect compounding burdens faced by women and girls. WASH-related responsibilities, for instance, often fall disproportionately on women, with the pandemic amplifying the demand for water and hygiene as part of non-pharmaceutical interventions for COVID-19 (20). At the same time, school closures and economic strain disrupted access to education and reproductive health services, placing adolescent girls at greater risk of pregnancy, early marriage, and permanent school dropout. These household-level disruptions, in turn, reinforced cycles of poverty and inequality.

WASH services were among those urgently required during COVID-19 to prevent transmission and maintain basic hygiene. SDG 6 calls upon the world to “ensure availability and sustainable management of water and sanitation for all” by 2030 [ (3): 14]. Several targets are then defined to achieve universal and equitable access to safe and affordable drinking water (SDG 6.1) and achieve access to adequate and equitable sanitation and hygiene, including ending open defecation (SDG 6.2). Special attention is given to the needs of women and girls. There is also a call to improve water quality (SDG 6.3). However, the advent of COVID-19 witnessed a pushback in progress as in some instances basic services came to a complete halt.

Compounding the strain on households was what has been described as a “pandemic within a pandemic” the rise in teen pregnancies and associated school dropouts. Mitana and Wendo (21) highlight that although COVID-19 affected everyone, “adolescent girls were the most affected due to socio economic barriers, further compounding their social and emotional well-being and sexual and reproductive health”. The work reveals a lack of systemic support at both at school and family/community levels. Musa et al. (22) found that early marriages and teenage pregnancies were significant, if often unspoken, consequences of the pandemic in Nigeria. Part of the aggravating circumstances were school closures during lockdowns and gender-based violence at home that forced other teens into marriages. However, all blame cannot be associated with the pandemic, as the practice of early marriages remains high in the country. Of interest are few perspectives raised by Rahiem (48) from a similar study in the province of Nusa Tenggara Barat province of Indonesia. The authors notice that teenagers got married because they thought and believed the marriages were an escape route “from schoolwork, house chores, and the stress and boredom of studying and staying at home during the pandemic” (Ibid.: 1).

### 2.2 Women responsibilities at home under COVID-19 lockdown

Coyle et al. (13) survey 280 families in 2020 and another 199 families more than a year later to generate insights on (1) pre-pandemic vs. current work-family conflict, (2) division of labour and schooling, and (3) children's daily activities. Findings from the first survey reveal mothers having increased work-family conflicts. Family had an impact on work as mothers took on primary responsibilities of children's education at home, while the girl child spent more time of house chores and also educating their younger siblings. This brought mental health challenges. During the second survey, stress levels for mothers subsided as they were working less from home, with children largely returned to face-to-face schooling. However, daughters were now spending more time caring for siblings compared to the early days of the pandemic. They were, however, also spending less time on chores, overall. The authors recommended that to mitigate the scenarios presented, governments had to consider policies alleviating the burden through paid family leave.

Women in academics became a big subject of research during COVID-19 (9, 10, 23). Power (23) acknowledges that while these women were already engaged in most of the unpaid care work in the world before COVID-19, this burden increased drastically during the pandemic. Similar findings emerged from India (10). To this end, without pro-active interventions from organised government, labour, civil society development partners, and other stakeholders (23), women and their families will be impacted for many more years to come. Such prolonged negative impacts will witness the 2030 Agenda for Sustainable Development in its SDGs and targets thrown out through the window. As this paper was being finalised, the world was in its 9th year of SDGs implementation, with 2030 coming ever closer.

Coming back to South Africa, Ronnie et al. (9) researched 2,029 women academics from 26 institutions of higher education The key burden that emerged was workload pressure. There were also issues regarding the provision of resources, top-down communication, with mistrust and limited support adding to the list. There was a misalignment of expectations between academics and their employers. Using the same data set, Walters et al. (49) went further to focus on the impact of COVID-19 on scholarly productivity of women academics While women are already underrepresented across the entire scholarly productivity spectrum, from peer review to publication, the pandemic resulted in further declines. Similar sentiments are expressed by Ucar et al. (24), that reminds us of the gender gap that has existed in academia, which expanded further during the pandemic.

In search of the causes of the decline, Walters et al. (49: 1) discovers that this was caused by “a dramatic increase in teaching and administrative workloads, and the traditional family roles assumed by women while ‘working from home”. Among the variables that resulted in a productivity decline were having younger (children under 6 years) or multiple dependents at home, home schooling and comforting anxious children. Therefore, the two at home responsibilities with the highest impact were childcare and assisting with schoolwork. Apart from childcare that was ranked first (accounting for 74.6% of the responses) and schoolwork (with 68.8%), housework came in 3rd at 66.8%, food preparation came in 4th with 58.9%, and getting supplies was 5th with 44.8% responses. Elsewhere outside of South Africa, parent-child conflicts were observed during homeschooling, adding to levels of stress (25).

While existing literature has extensively examined the gendered implications of COVID-19, including women's increased domestic responsibilities, disruptions in education, and the exacerbation of vulnerabilities such as teen pregnancies and early marriages, significant research gaps remain. First, much of the current scholarship either addresses these issues in isolation or focuses on high-income or Global North contexts, with limited integrative analyses capturing the interlinkages between WASH (water, sanitation, and hygiene) challenges, domestic care burdens, and adolescent vulnerabilities within low-resource, Southern African settings. Second, few studies explore how these intersecting stressors simultaneously unfolded at the household level during the COVID-19 lockdowns, especially through a gender-responsive lens that connects macro-level development goals (such as SDG 6) with lived experiences on the ground. This study responds to these gaps by providing a context-specific, intersectional analysis of household-level pressures experienced by women and girls in South Africa during the pandemic.

## 3 Materials and methods

**Figure 1:**
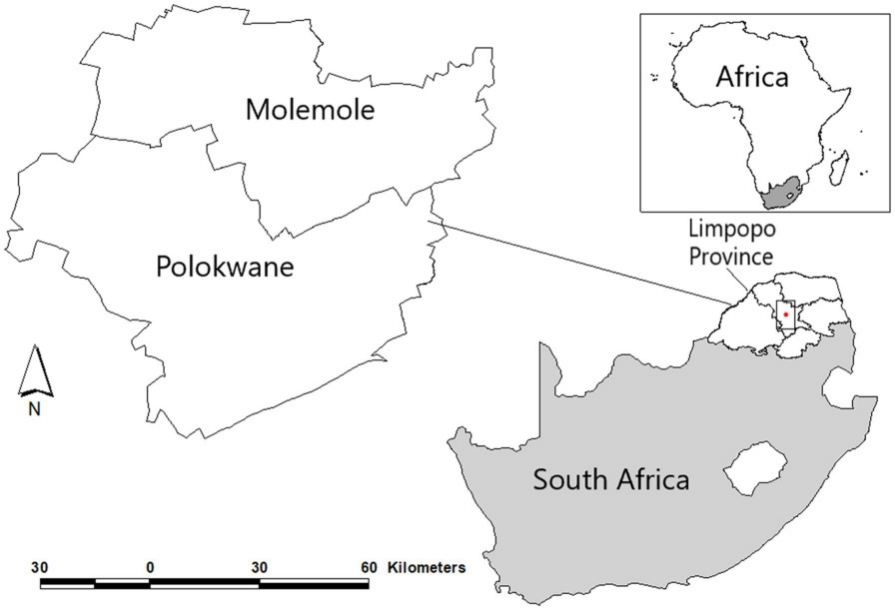
Location of the study area.

Research is generally informed by the 2030 Agenda for Sustainable Development (2030 AfSD) and its 17 interwoven SDGs and Paramita et al. (26) broader conceptualisation of gender that goes beyond binary biological sex. Drawing the 2030 AfSD (3), of particular importance were SDG 5 (Gender), SDG 4 (quality Education) and SDG 6 (Water and Sanitation). With this in mind, the paper sought to investigates the impact of COVID-19 lockdowns on women and the girl child's roles and responsibilities at the household level in South Africa's Limpopo Province. The focus was on two local municipalities that are part of the Capricorn District Municipality, namely: Molemole and Polokwane (Figure 1). These municipalities were purposefully selected based on guidance from the Limpopo Provincial Government to capture both urban and rural dynamics within the province. Polokwane, as the provincial capital, represents a more urbanised and economically active setting, whereas Molemole reflects a predominantly rural context with limited access to basic services. This diversity allowed the study to assess the differentiated impacts of COVID-19 lockdowns across contrasting socio-economic environments.

To continue with the research, an ethics clearance was granted by the Limpopo Provincial Research Ethics Committee on July 20, 2022, for Research Project No. LPREC/35/2022. The Limpopo Provincial Research Ethics Committee (LPREC) was registered with the National Health Research Council (NHREC) Registration Number REC-111513-038. Two surveys were also pitched. These included a household survey and a complementary teacher survey. Survey instruments were developed through a consultative process involving gender studies experts, local educators, and community representatives. Questions were informed by existing literature on gender roles, COVID-19 impacts, and household dynamics, as well as previously validated instruments such as the UNICEF COVID-19 Rapid Assessment tool (27), which were adapted for the local context. Both surveys were reviewed by three independent subject matter experts to ensure content validity, assessing relevance, cultural appropriateness, and clarity. An internal consistency was measured using Cronbach's alpha. Acceptable values above 0.7 confirmed sufficient internal reliability.

Internal reliability was further assessed for the main multi-item constructs included in the household and teacher survey instruments. Cronbach's alpha values above 0.7 were recorded across all constructs, confirming adequate internal consistency. Specifically, three sets of scales were tested: (1) a household responsibilities scale comprising items on time allocation to various domestic activities such as food preparation, childcare, household chores, and emotional support, each measured using a five-point Likert scale ranging from 1 = decreased drastically to 5 = increased drastically; (2) a WASH service reliability scale that captured perceptions of water adequacy, source reliability, and sanitation access, measured on a four-point Likert scale (1 = very unreliable to 4 = very reliable); and (3) a gendered division of labour perception scale assessing agreement with statements regarding men's and women's roles in household tasks, rated on a five-point agreement scale (1 = strongly disagree to 5 = strongly agree). The Cronbach's alpha coefficients for these scales ranged from 0.71 to 0.84, confirming acceptable levels of reliability for all constructs used in the study.

A census sampling approach was used, targeting all accessible households and public schools within the identified wards of both municipalities. The goal was to include the entire available population within each targeted area, thereby maximising coverage and minimizing sampling bias. For the household survey, the inclusion criteria specified adults aged 18 years and older residing in the household at the time of the survey. Questions specific to the perceived experiences of women during the COVID-19 pandemic were included in the survey, as the study focused on gendered impacts. The inclusion criteria required participants to be residents of the selected local municipalities and aged 18 or older. While women were the primary respondents for questions related to gendered experiences, if no adult woman was available or willing to participate, any other adult household member knowledgeable about household dynamics was interviewed. Households were excluded if no adult respondent was present or if residents declined participation.

This approach resulted in 4,571 completed household surveys and 226 teacher surveys conducted between October 2022 and March 2023. While the sample may not be statistically representative of the broader Limpopo Province or the entire Capricorn District, it is reflective of the population structures within the selected wards. Demographic characteristics of the respondents were compared with the 2016 South African Community Survey and the 2022 mid-year estimates from Statistics South Africa. These comparisons confirmed general alignment with local population trends, including household composition, age distribution, employment status, and gender, although there was a slight overrepresentation of female-headed households, likely a result of the study's focus on gendered roles during the pandemic. Despite the high response rate (approximately 99.9%), the study acknowledges the potential for non-response bias and sampling errors. Certain households may have been unavailable or unwilling to participate, and this could have introduced minor biases related to household characteristics such as income, gender composition, or access to services. Efforts were made to minimize this through multiple revisits and the use of local enumerators familiar with the communities.

The household survey included sections on household demographics, water access, sanitation, education disruptions, and the gendered division of labour during lockdowns. The teacher survey focused on perceptions of learners’ well-being, educational setbacks, and observed shifts in gendered responsibilities among students. The surveys were uploaded to the QuestionPro platform, a facility that permits both on and offline administering of surveys. This was critical as the Limpopo province is known to be predominantly rural (28) with challenges internet connectivity.

Descriptive summary statistics were generated to determine the characteristics of the sample, with counts and percentages reported. To test bivariate associations, the study employed a One-way Analysis of Variance (ANOVA) to examine if there were any noteworthy differences among age groups and household sizes concerning the time women spent on various responsibilities during the lockdown. Before conducting the ANOVA, we assessed the assumptions associated with this statistical test to ensure the appropriateness of the data. Specifically, normality and homogeneity of the data were examined using the Shapiro–Wilk test and Levene's test, respectively. The data exhibited mild violations of normality and homogeneity. However, since these violations were not significant, we proceeded with the one-way ANOVA test. To identify age groups and household sizes with significant differences, we employed the Tukey HSD *post-hoc* test, reporting unadjusted *p* < 0.05 values. All analyses were performed using JMP Pro SAS for Windows, version 16.2.0.

## 4 Presentation of findings

The findings are presented in four sub-sections. The sub-sections include general demographics of the responding women, potential pressure points from lack of basis services like WASH, plight of the girl child, and the impact of COVID-19 on women at home. Each of these sub-sections will now be considered in turn.

### 4.1 The demographics

The demographic information presented pertains to the household survey. During the survey, a total of 6,172 households were approached, with 4,571 households having participants who completed the survey. This gave a very high completion rate of about 99.9%. This high return rate was largely attributed to the face-to-face administration of the survey by 30 trained field workers. On average, the survey took 22 min.

In terms of gender distribution among respondents, 66.3% were female, 33.7% were male and a minimal 0.1% chose not to disclose their gender. The bias towards females aligns with the focus of this article on explaining how COVID-19 in Limpopo province affected women and girls. Regarding the level of education, most of the respondents (39.7%) had completed secondary education, and a significant number had tertiary education. Moreover, 60.9% of the respondents identified as household head. The age cohorts were almost evenly spread with close to 33.1% aged 60 and above. From all other age groups, the percentage share ranged from 15.7% to 18.2%. Marital status varied, with 32.5% of the respondents in marriage, 17.2% windowed, 47.5% single, 2.5% divorced and 0.4% choosing not to disclose their marital status.

Household size analysis revealed that the majority had between 3 and 4 people, closely followed by 5–6 people. There were also significant households with seven or more people (20%), while those with 1–2 people accounted for 16.9%. Examining employment status, 48.5% of women respondents were unemployed, 30.5% were in retirement and/or pensioners, 5.7% were full-time workers, 5.4% were part-time workers, 3.9% were students and 0.5% chose not to disclose their employment status. Finally, with respect to household income, 75.8% of respondents reported a monthly household income below R4,500^1^. The subsequent income brackets were R4,501–R9,000 (9.3%), R9,001–R13,500 (3.4%) and R13,001–R18,000 (1.3%), and over R18,001 (2.2%), while 7.9% were not sure of their monthly household income. Further details are presented in Table 1.

**Table 1:** Socio-demographic characteristics of surveyed households.

Characteristics	N (%)
Position held in the household	
Household head	1,535 (49.7)
Spouse	584 (18.9)
Daughter	749 (24.3)
Relative	79 (2.6)
Son	15 (0.5)
Other	126 (4.1)
Age (in years) groups	
18–29	511 (16.5)
30–39	562 (18)
40–49	495 (16)
50–59	498 (16)
60–69	1,010 (33.1)
Not disclosed	12 (0.4)
Education levels	
No formal education	511 (16.5)
Completed primary education	873 (28.3)
Completed secondary education	1,225 (39.7)
Tertiary education	457 (14.8)
Not disclosed	22 (0.7)
Marital status	
Married	1,003 (32.5)
Single	1,465 (47.4)
Divorced	77 (2.5)
Widowed	529 (17.1)
Not disclosed	14 (0.5)
Employment status	
Full-time employment	173 (5.6)
Part-time employment	166 (5.4)
Self-employed	177 (5.8)
Student	119 (3.9)
Unemployed	1,498 (48.7)
Retired	44 (1.4)
Pensioner	897 (29.2)
Unable to work	14 (0.5)
**An estimate of household income per month**	
<R 4 500	2,341 (75.8)
R 4 500–9 000	289 (9.4)
R 9 001–13 500	105 (3.4)
R 13 501–18 000	40 (1.3)
R 18, 001+	69 (2.2)
I am not sure	244 (7.9)
Household size	
1–2	521 (17)
3–4	1,065(34)
5–6	884(29)
7+	618(20)

The table shows a predominance of female respondents and medium-sized households, providing context for understanding gendered patterns in domestic responsibilities.

### 4.2 The potential pressure points

**Figure 2:**
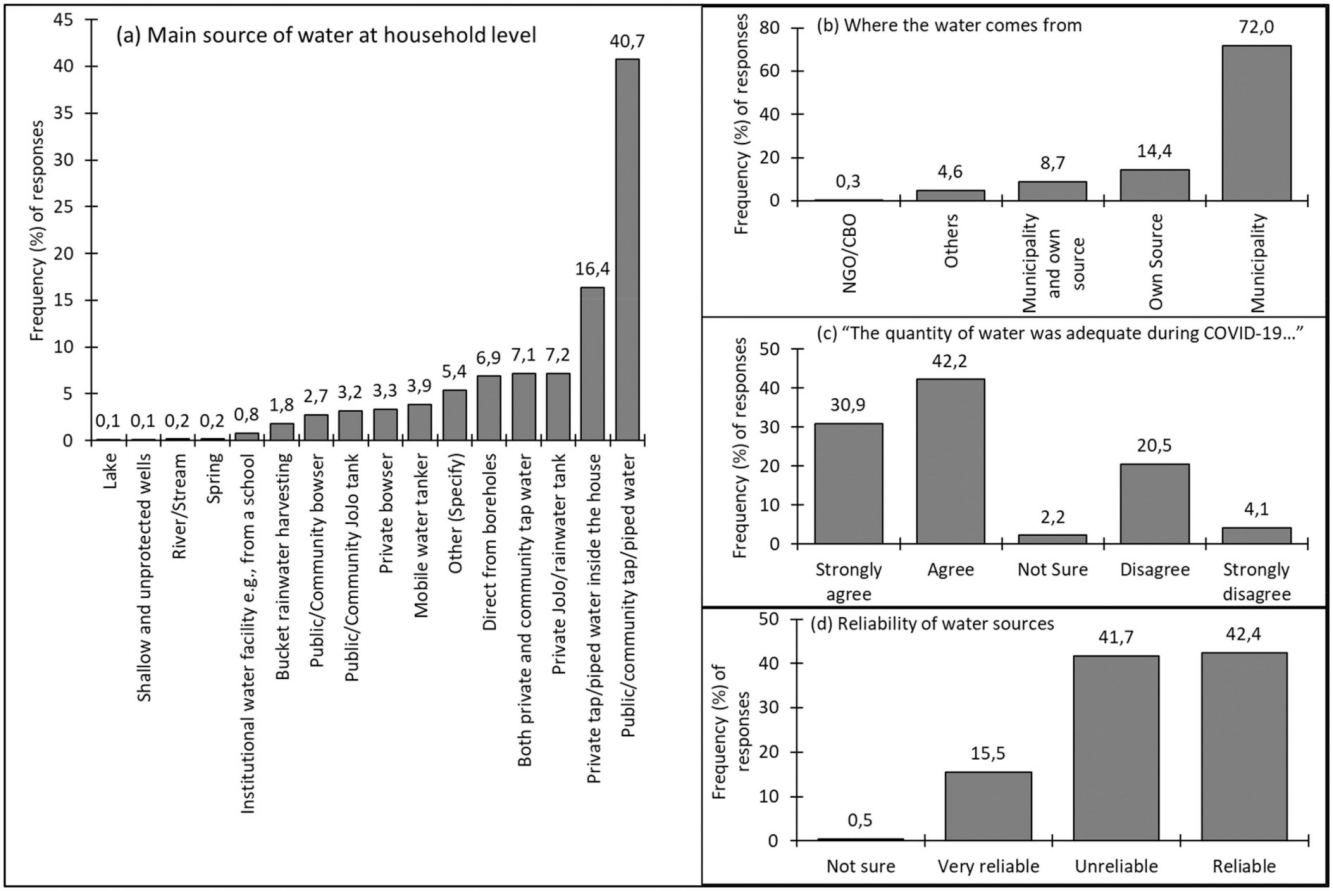
Distribution and reliability of domestic water sources during the COVID-19 lockdown. Although municipal water was the dominant source, over 40% of households reported inadequate or unreliable supply, revealing WASH service gaps that intensified women's daily labour burdens.

Potential pressure points impacting women's roles and responsibilities at the household level were investigated. Among such matters were WASH, living with patients with comorbidities, and food supplies. WASH remained at the centre of non-pharmaceutical interventions for COVID-19. To this end, it was necessary for this work to determine water sources, availability, and reliability at the household level. When asked about their main sources of domestic water during the pandemic, most of the respondents (40.7%) indicated that they were using tap water from the community. Following this, 16.4% mentioned having tap water in their own households, with the remaining portion shared by 13 other sources, and 5.4% of respondents who indicated “other sources” (Figure 2A). The investigation also revealed that the municipality served as the main source for 72.0% of the respondents, with private sources accounting for 14.4%, a combination of the two at 8.7%, and other sources taking up the remining percentage (Figure 2B). Generally, the respondents strongly and/or agreed that the water quantities were adequate (73.1%), with 24.6% of the respondents indicating the quantity was not adequate. Only 2.2% of the women surveyed were not sure (Figure 2C). Asked to establish the main sources of water affected by the pandemic, the top two main ones highlighted in Figure 2A were said to have been impacted, with 53.5% of the surveyed women confirming this.

Furthermore, a significant number of the respondents (41.7%) also revealed that the water sources they used were unreliable, while 42.4% found their sources to be reliable. Despite 15.5% of the women indicating that their water sources were very reliable, a minimal figure (0.5%) could not ascertain the reliability of their water source (Figure 2D).

Linked to WASH was the issue of ablutions and/or toilet facilities. Approximately 59.8% of respondents reported that their households use private and outside the home toilet facilities. Following this, 26.3% used private and inside the house toilets, and 8.44% used public/community toilets. Additionally, 3.3% of respondents used both community and private toilet facilities. Those who did not specify comprised 2.2% of the respondents.

### 4.3 Plight of the girl child and additional challenges faced by women

**Figure 3:**
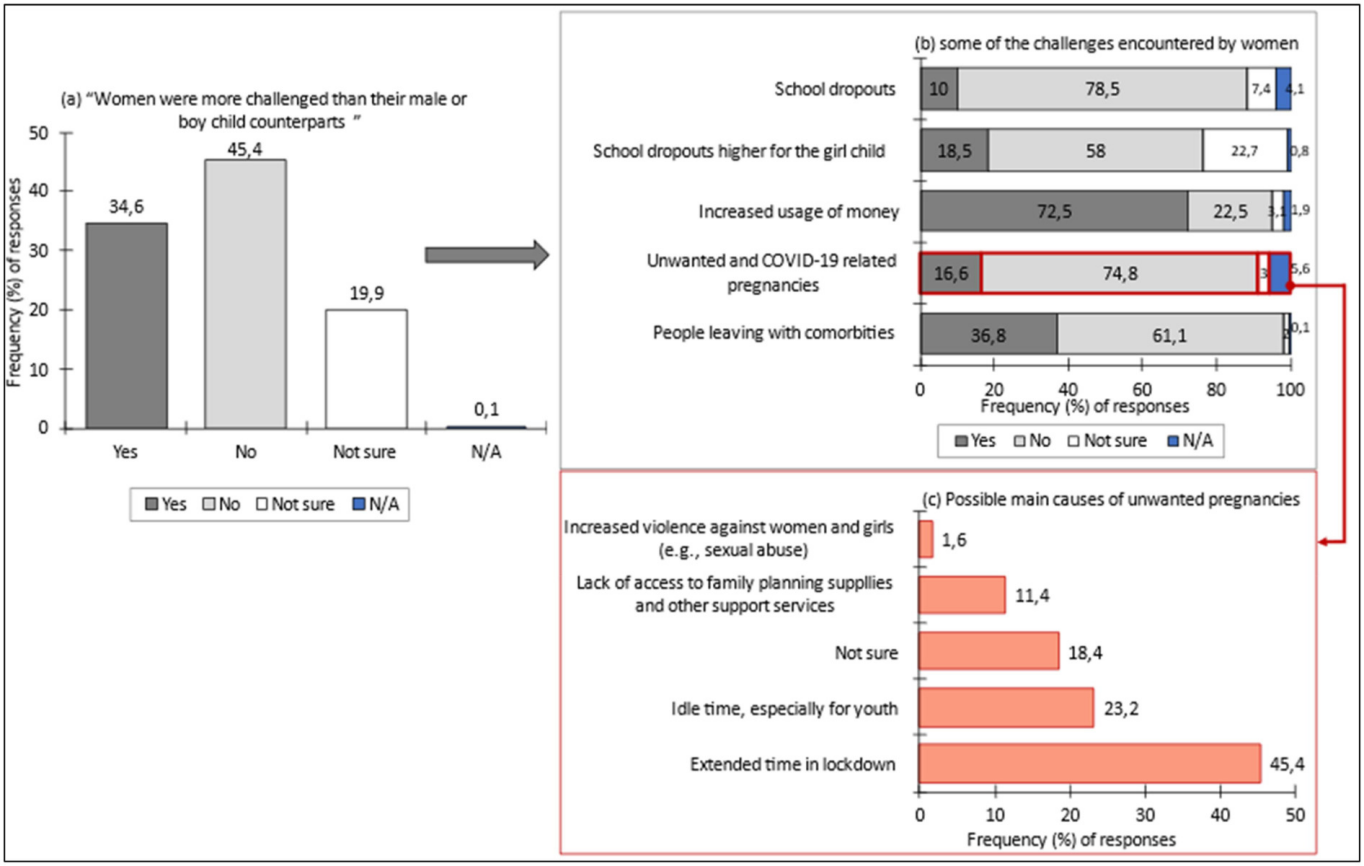
**(a–c)** perceived impact of COVID-19 on the girl child and women. Data for **(a,b)** are derived from the household survey; illustrate heightened domestic workloads and psychosocial stress experienced by women during lockdowns, while panel **(c)** drawn from teacher survey responses, reveals educators’ observations of increased vulnerability and reduced educational engagement among girls. Collectively, the results demonstrate how pandemic restrictions reinforced pre-existing gender inequalities in care, education, and well-being.

Numerous studies have documented that the COVID-19 pandemic disproportionately affected women and girls, particularly in terms of mental health, unpaid caregiving burdens, and increased vulnerability to socio-economic stressors (15, 29, 30). In the present survey, respondents were asked whether they believed women and the girl child were more adversely affected by COVID-19 than men or boys. A total of 34.6% of women affirmed this view, while 45.4% did not. The remaining 20.0% expressed uncertainty (Figure 3A). Additionally, the continuity of education for the girl child was also a point of vulnerability. When respondents were asked to assess whether there was an increase in school dropouts for girls during the pandemic, 18.5% confirmed this, while 58% disagreed and 22.7% were unsure (Figure 3B). Addressing the issue of unwanted and COVID-19 related pregnancies in households, close to 75% (*n* = 2,303) of respondents reported not facing such incidents. The top three cited causes for these pregnancies were the extended time in lockdown (45.4%), idle time, especially for the youth (23.2%), and the lack of access to family planning supplies and support services (11.4%) (Figure 3C).

**Figure 4:**
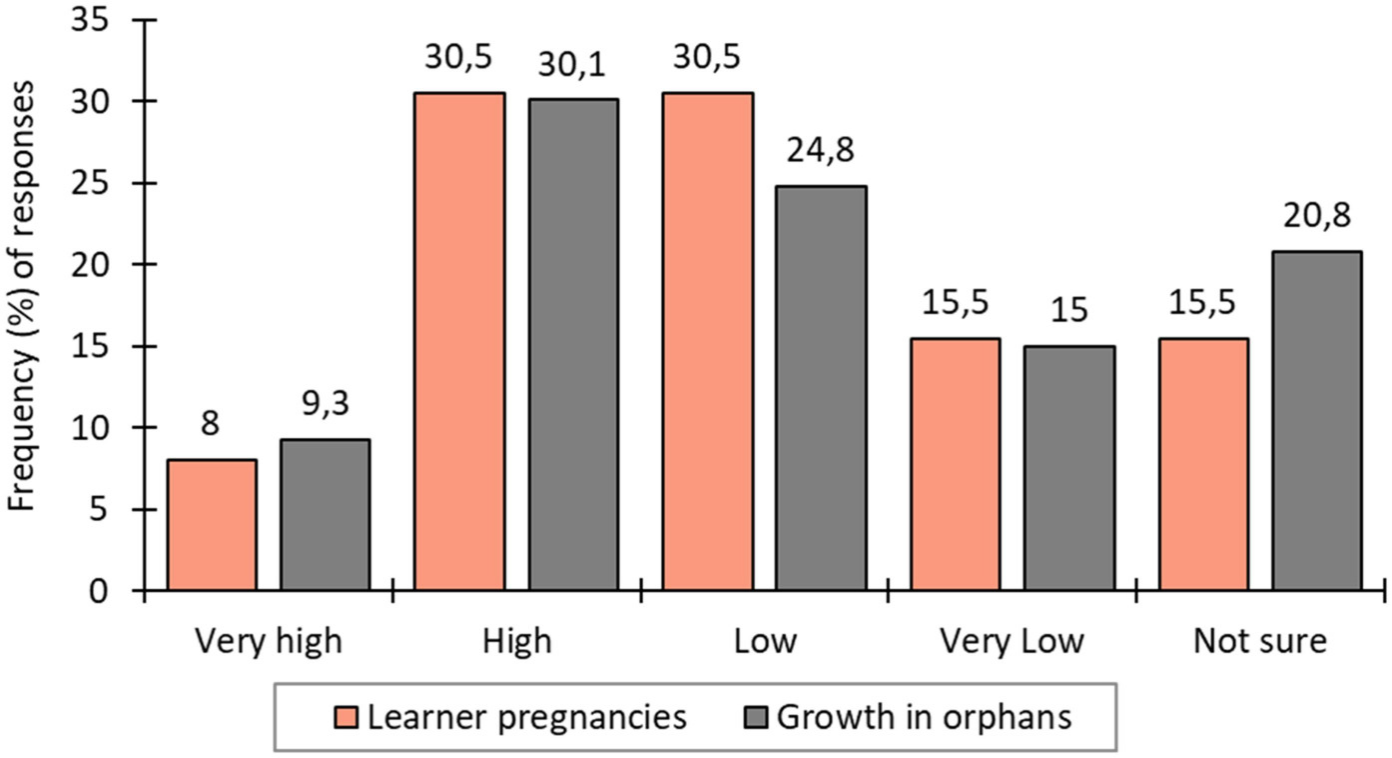
Teachers’ reports of learner pregnancies and increases in the number of orphans during the pandemic period.

To triangulate the findings, a question was asked in the teacher survey (*n* = 226) regarding the extent of learner pregnancies in schools during COVID-19. About 38.5% of respondents noted high to very high incidences, 46.0% observed low to very low incidences and 15.5% were uncertain (Figure 4). The teacher survey further indicated that 39.4% observed a high to very high growth in orphans, while 39.8% reported low to very low growth and 20.8% were uncertain about such growth (Figure 4).

Regarding comorbidities, 36.9% of the respondents indicated the presence of someone with comorbidities, while 61.1% did not. The remaining 2% were unsure. Living with people with comorbidities remained a traumatic experience, as research work had revealed high death rates due to COVID-19 in such people. In fact, hypertension, cardiovascular disease, and diabetes remained the most common comorbidity for those who died of COVID-19, and patients who had two or more comorbidities were the most vulnerable (31). Apart from dealing with patients with comorbidities, households had to deal with orphans.

### 4.4 Impact of COVID-19 on women at home in Limpopo

The question of how women spent their time in the household during COVID-19 remains a central topic of investigation in this paper. It emerged that during the lockdown, the time allocation of women towards various activities underwent significant shifts, reflecting the multifaceted impact of the pandemic on their daily responsibilities. Notably, a considerable proportion reported increased time spent on food planning and preparation (26.1% drastically increased, 52.5% increased) and household chores (24% drastically increased, 58% increased). Baby and child minding also saw notable changes, with 20% experiencing a drastic increase and 51% reporting a general increase. From the seven main parameters investigated regarding the responsibilities of women at home under lockdowns, there were noticeable drastic increases and/or increases in household chores (ranked 1st), meal planning and preparation (2nd), providing social and emotional support (3rd), baby and child minding (4th) and home schooling and/or school homework (5th). The continuity of education for girls was a point of vulnerability, as 18.7% confirmed an increase in school dropouts. Tending to the sick and providing social and emotional support showed varying degrees of impact, while work-related activities saw changes of 3.3% in a drastic increase and 15.4% in a general increase. The results are shown in Table 2.

**Table 2:** Evaluation of responsibilities and time spent by women at home during lockdown, based on household survey data. Data reported N(%).

Time spent by women on the following during lockdown	Increased drastically	Increased	Decreased	Decreased drastically	Not sure	N/A
Food/mean planning and preparation (R1).	807 (26.1)	1,620 (52.5)	237 (7.7)	26 (0.8)	105 (3.4)	292 (9.5)
Household chores (R2)	739 (24)	1,775 (58)	193 (6)	19 (1)	104 (3)	248 (8)
Baby and child minding (R3)	624 (20)	1,568 (51)	85 (3)	17 (1)	76 (2)	714 (23)
Home schooling and/or school homework (R4)	507 (16.5)	1,360 (44.1)	220 (7.1)	40 (1.3)	117 (3.8)	837 (27.2)
Tendering the sick (R5)	303 (9.8)	637 (20.7)	90 (2.9)	38 (1.2)	115 (3.7)	1,896 (61.6)
Providing social and emotional support (R6)	512 (16.6)	1,720 (55.9)	98 (3.2)	35 (1.1)	114 (3.7)	599 (19.5)
Work-related activities (R7)	101 (3.3)	473 (15.4)	246 (8)	68 (2.2)	112 (3.6)	2,078 (67.5)
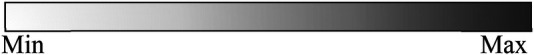						

The table highlights a substantial rise in time devoted to childcare, cooking, and cleaning, confirming that women's unpaid domestic workload intensified markedly during the lockdown period.

**Figure 5:**
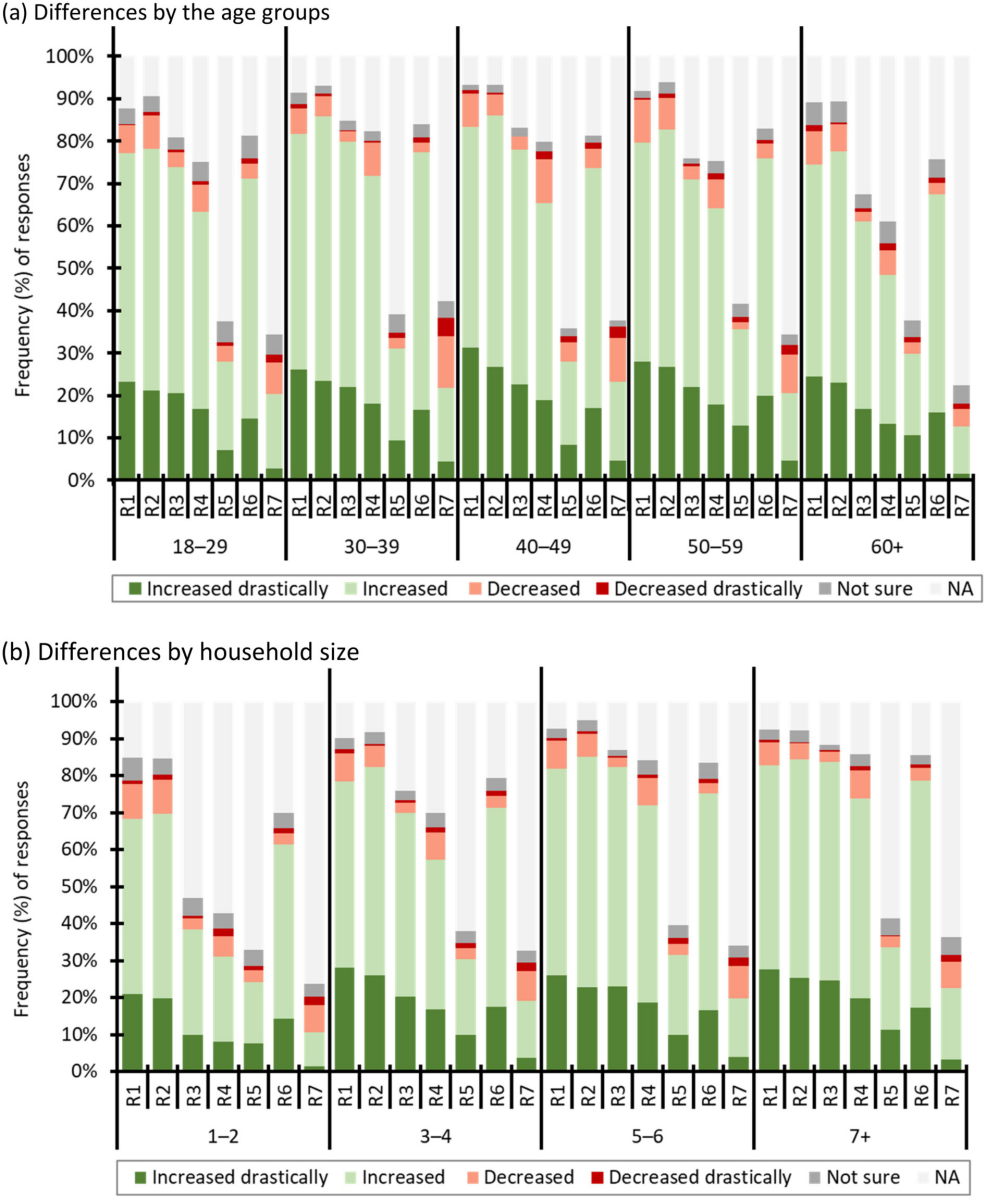
**(a,b)** assessment of women's time allocation across household responsibilities. Panel **(a)** shows variations by age cohort, indicating that older women spent proportionally more time on caregiving and domestic tasks, while panel **(b)** demonstrates that women in larger households faced heavier workloads. These findings confirm that both age and household composition significantly shaped the gendered distribution of unpaid labour during lockdowns.

As shown in Figure 5, the way women spent their time during the lockdown varied depending on the size of their households (*F* = 6.87, *p* = 0.001) and the different age groups of women (*F* = 7.937, *p* < 0.0001), with post-doc results indicating distinctions across various age groups and household sizes (Table 3).

**Table 3:** Results of tukey HSD *post hoc* tests comparing mean time allocation across seven household responsibilities.

Parameter	Level	-Level	Mean Diff.	SE	Sig.
Age	60+	18–29	82.69	27.77	.000
		30–39	74.69	27.77	.001
		40–49	85.88	27.77	.000
		50–59	85.36	27.77	.000
Household size	3–4	1–2	90.96	23.35	.001
		7+	74.50	23.35	.009

The analysis identifies statistically significant pairwise differences, indicating that certain tasks, particularly childcare and meal preparation, required significantly greater time commitments than others during the lockdown.

Table 3 revealed a notable trend where women aged 60 and above spent significantly more time on all the household responsibilities listed in Table 2 compared to women in other age categories. Similarly, a comparable trend was observed among women residing in households with 3 to 4 people.

Table 4 complements the findings in Figure 5 by summarising the statistical results for the association between each responsibility, age, and size of households, including significant *post-hoc* test results denoting specific age cohorts’ comparisons (Table 5). The results indicate significant variations in time allocation for responsibilities across different age cohorts and, to a certain extent, household sizes. Specifically, age is a significant factor for most responsibilities, while household size plays a role in some instances. For all the responsibilities (R1 to R6), age significantly influences time allocation as indicated by with significant *p*-values (for all *p* < 0.05). However, for R7, age does not seem to play a significant role, with a higher *p* = 0.348. Additionally, the influence of household size is less consistent, showing significance in some cases (e.g., R1 and R2) but not in others.

**Table 4:** Summary of ANOVA results examining time allocation to household responsibilities across age groups and household sizes, derived from household survey data.

Responsibility/activity	Dependent variable	Sum of Squares	df	F	Sig.
Food/mean planning and preparation (R1)	Age	32,853.5	4	5.840	.003
	Household size	30,851.7	3	3.340	.048
Household chores (R2)	Age	32,893.9	4	4.712	.008
	Household size	30,814.5	3	2.440	.104
Baby and child minding (R3)	Age	32,853.5	4	4.078	.014
	Household size	30,851.7	3	1.358	.293
Home schooling and/or school homework (R4)	Age	32,985.0	4	3.556	.024
	Household size	30,851.7	3	1.609	.229
Tendering the sick (R5)	Age	32,853.5	4	2.917	.047
	Household size	30,851.7	3	3.163	.056
Providing social and emotional support (R6)	Age	32,853.5	4	4.790	.007
	Household size	30,935.5	3	3.063	.060
Work-related activities (R7)	Age	32,853.5	4	1.183	.348
	Household size	30,851.7	3	2.927	.067

The results show significant effects (*p* < 0.05) of both age and household size, suggesting that older women and those in larger households carried heavier domestic and caregiving burdens.

**Table 5:** The results of *post hoc* test for multiple comparisons of responsibilities categorized by age, based on household survey data (only statistically significant differences are presented).

Dependent variable	Level	-Level	Mean Diff.	SE	Sig.
Food/mean planning and preparation (R1)	60+	18–29	82.67	21.65	.009
		30–39	74.67	21.65	.019
		40–49	85.33	21.65	.006
		50–59	85.33	21.65	.006
Household chores (R2)	60+	18–29	82.67	24.12	.016
		30–39	74.67	24.12	.040
		40–49	86.0	24.12	.015
		50–59	85.33	24.12	.016
Baby and child minding (R3)	60+	18–29	82.67	25.91	.033
		30–39	74.67	25.91	.063
		40–49	85.83	25.91	.026
		50–59	85.33	25.91	.027
Home schooling and/or school homework (R4)	60+	40–49	86.00	27.80	.041
		50–59	85.5	27.80	.042
Providing social and emotional support (R6)	60+	18–29	82.67	23.89	.019
		30–39	74.67	23.89	.038
		40–49	85.83	23.89	.014
		50–59	85.33	23.89	.015

The results indicate that older women spent significantly more time on caregiving and household chores than younger cohorts, underscoring the influence of life stage on gendered divisions of labour during the lockdown.

To improve statistical transparency, 95% confidence intervals (CIs) were calculated for key proportions and mean differences reported in the survey data. For example, the estimated increase in school dropouts among girls (18.5%) had a 95% CI of [17.2–19.8], while the proportion of respondents reporting COVID-19–related teenage pregnancies (approximately 25%) had a 95% CI of [22.9–27.1]. In addition, effect sizes were computed for the ANOVA tests to quantify the magnitude of associations observed between age, household size, and time allocation across household responsibilities. Partial eta-squared (*η*^2^) values ranged from 0.03 to 0.06, indicating small to moderate effects according to Cohen's (50) criteria.

The *post hoc* Test did not reveal any significant differences between household sizes and all the responsibilities. However as indicated in Table 5, significant differences in mean values were observed across various age groups, indicating variations in time allocation for these responsibilities. Notably, the age group 60 + consistently showed higher mean differences when compared to younger age categories (18–29, 30–39, 40–49, 50–59) in these responsibilities, highlighting the influence of age on time spent. However, no statistical differences were found among the age groups for responsibilities R5 and R7.

## 5 Discussions

Findings from the survey show that the COVID-19 lockdown period was associated with changes in women's daily responsibilities, highlighting longstanding gender disparities in unpaid care and domestic work. The first edition of the South Africa COVID-19 Country Report (2021) highlights the significant gendered impact of the pandemic, with the term women appearing over 400 times and girl(s) 21 times (28). An entire chapter is dedicated to gender equality issues arising from the pandemic, revealing a disturbing trend of increased burden and trauma for girls. The report points to a higher incidence of COVID-19 infections and hospitalisations among girls aged 15–18, potentially linked to their disproportionate involvement in care responsibilities at home.

Our study found marked increases in women's time spent on household chores, meal preparation, childcare, and homeschooling during lockdown. In contrast, caring for the sick and engaging in work-related activities did not show similar increases. Age-specific analysis revealed that women aged 60 and above consistently spent more time on household duties, emphasizing the need for age-responsive policies that account for the differing capacities and roles of women across the life course. Additionally, time allocation varied with household size, highlighting the importance of context-sensitive approaches in policy design. Comparable findings have been reported elsewhere. For example, Chauhan (10) observed a stark gender divide in unpaid domestic work in India, with no men represented in the highest time-use categories. Approximately 5% of women spent 22–28 h per week on household chores (compares to 0% of men), 12.3% spent 29–49 h (vs. 2.3% of men), and nearly 9% of women reported spending over 50 h per week, a time commitment not reported by any male participant. These patterns were echoed in other countries: Hjálmsdóttir and Bjarnadóttir (32) describe how Icelandic women bore the brunt of social and emotional labour during the pandemic, while Rania et al. (33) observed in Italy how women, particularly mothers, shouldered the burden of reconfiguring shared living spaces and maintaining emotional support in quarantine scenarios. This emotional labour was often unreciprocated, even as women themselves required support, as Philpot et al. (34) documented in the United States.

In Limpopo province, meal planning and preparation were ranked second among women's increased responsibilities during lockdowns. Snuggs and McGregor (35) in a UK-based study, found that lockdowns impacted food choices and availability, requiring considerable time investment in logistics. Participants placed increased importance on health, weight control, and mood when selecting food, far above considerations like familiarity. Childcare and baby minding responsibilities also fell heavily on women. While Fodor et al. (36) found in Hungary that men increased their childcare contributions by 35 his was proportionally less impactful, as women already bore the majority of childcare responsibilities before the pandemic. In absolute terms, women's increased unpaid care work far outpaced men's, particularly among educated, middle-class urban women. The National Department of Planning, Monitoring and Evaluation (28) also reported that despite lockdowns providing more time for men to be with their children, caregiving remained primarily a female duty.

Homeschooling introduced another layer of stress, especially for women. With schools closed and online platforms unfamiliar to many, women often in dual roles as breadwinners and caregivers were overwhelmed. This is particularly relevant for the present study, where 60.85% of respondents came from female-headed households. Similar challenges were reported in Poland (37), where parents, particularly mothers, felt unequipped to homeschool and experienced heightened anxiety about their children's futures. In some cases, homeschooling led to work-family conflicts, with increased homeschooling hours among women correlating with higher alcohol consumption among couples.

The issue of unwanted pregnancies also warrants attention. Although approximately 75% of respondents reported no incidents, the associations observed with extended lockdown periods, idle time, and limited access to family-planning supplies indicate areas requiring policy attention. Our study indicates that 38.5% of teachers reported observing learner pregnancies during the pandemic period. In Gauteng, the number of births among girls under 18 rose by 60% between April 2020 and March 2021, with more than 23,000 girls under 18 years, 934 of them under 14 years giving birth (38). Barron et al. (39) similarly found that rural provinces such as Limpopo, Mpumalanga, and Eastern Cape experienced higher rates of teenage pregnancy than more urban areas. This trend is consistent with findings from Ghana, where Mohammed (40) noted increases in child marriage linked to poverty and school closures. Some girls expressed a desire to marry, citing household chores as overwhelming. Phukula et al. (41) confirmed that such patterns were not isolated, with similar trends emerging in Botswana and other parts of the world.

An increase in reported orphanhood (39.4% of teachers noting high to very high growth during the pandemic) indicate the need for strengthened social welfare responses. Policymakers should focus on aiding families facing this situation, ensuring the well-being and support of orphaned children (42, 43). Another concerning issue is the reported increase in school dropouts, particularly among girls, poses challenges to achieving Sustainable Development Goals 4 and 5. SDG 4 aims to ensure inclusive and equitable quality education and lifelong learning opportunities for all, with Target 4.1 seeking full completion of primary and secondary education by 2030 [ (3): 14]. School dropouts often went hand-in-hand with child marriage and its associated consequences (40).

Access to clean water also emerged as a critical issue. Respondents’ reports of unreliable water sources highlight constraints that may have limited households’ ability to comply fully with WASH (Water, Sanitation and Hygiene) -related COVID-19 prevention measures (44, 45). Despite global emphasis on hygiene, many informal settlements in the global South lacked adequate WASH infrastructure. Howard et al. (46) noted that hygiene became central in the pandemic response, but billions lacked piped water access. Mieth et al. (47) documented the behavioural shift to frequent handwashing, corroborated by Lin et al. (51) who found that online searches for “wash hands” were associated with slower spread of the virus, unlike “face mask,” which showed no such correlation. In South Africa, the National Department of Planning, Monitoring and Evaluation [ (28): 304] acknowledged that WASH challenges existed even before COVID-19 but were greatly exacerbated by the pandemic. In 2019, about 12% of the population lacked access to a basic water supply, and 21.3% had no access to basic sanitation. These figures likely underrepresent the challenges in rural provinces like Limpopo. Media footage during the pandemic often showed women and girls as the primary collectors of water from tanks and delivery tankers, highlighting how the pandemic period represented a dual challenge for the girl child, that was marked both by heightened health risks and by widening gender inequalities.

### 5.1 Limitations of the study

Although a census sampling approach was used to include all accessible households and schools within the selected wards, the findings cannot be generalized to the entire Limpopo Province. The sample reflects local community characteristics rather than those of all municipalities, and some non-response bias may have occurred despite mitigation through repeated visits and local enumerators. As the study relied on self-reported data, responses may be influenced by recall or social desirability bias. Contextual differences in service delivery and socio-economic conditions may also have affected results. Finally, the cross-sectional design captures a specific phase of the COVID-19 lockdown, limiting temporal comparisons. The findings should therefore be viewed as indicative of trends within the sampled areas, and future studies should use larger, representative samples to enhance external validity.

## 6 Conclusions

Our investigation into potential pressure points affecting women's roles and responsibilities at the household level amidst the COVID-19 pandemic revealed critical concerns. The central focus on Water, Sanitation, and Hygiene (WASH) highlighted a reliance on community tap water supplied by the municipality, although a notable proportion faced challenges with unreliable water sources. The impact on the girl child was pronounced, with varying opinions on whether women and girls disproportionately bore the pandemic's burdens, manifested in disruptions to education, reported school dropouts, and concerns about unwanted pregnancies. Living with individuals with comorbidities posed challenges, accentuated by the heightened risk of severe outcomes, while the increase in orphans within households added complexity. Survey results revealed significant shifts in women's time allocation during the pandemic period, particularly for those aged 60 and above, emphasizing the influential role of age in shaping household responsibilities. Addressing these multifaceted issues is imperative for informed policy interventions that support the resilience and well-being of women and vulnerable populations during health crises. The findings suggest that the pandemic period may have slowed progress toward achieving the 2030 Agenda for Sustainable Development and the 17 SDGs. Specific SDGs to note include SDG 3 (Health and Wellbeing), SDG 4 (Quality Education), and SDG 5 (Gender Equality).

These findings hold crucial policy implications. Ensuring reliable and accessible WASH services is imperative for household resilience during health crises. Addressing the vulnerability of the girl child in education and health services requires targeted interventions. Additionally, the high incidence of learner pregnancies in schools emphasizes the need for comprehensive sexual education programs. Efforts to maintain access to family planning resources should be prioritized to mitigate unintended pregnancies. Support systems for households with comorbidities and orphans are essential. Policies should strengthen WASH infrastructure, sustain education continuity, and ensure access to reproductive health services. Social and emotional support systems need enhancement, especially for households dealing with comorbidities and orphans. Additionally, age-sensitive policies should be devised, recognizing the unique challenges faced by older women.

## Data Availability

The raw data supporting the conclusions of this article will be made available by the authors, without undue reservation.

## Footnotes

^1^The exchange rate averaged $1=ZAR 18.70 in January 2023.
